# Amelioration of Liver Injury by Continuously Targeted Intervention against TNFRp55 in Rats with Acute-on-Chronic Liver Failure

**DOI:** 10.1371/journal.pone.0068757

**Published:** 2013-07-16

**Authors:** Yumin Xu, Hui Wang, Shishan Bao, Fazal Tabassam, Wei Cai, Xiaogang Xiang, Gangde Zhao, Haiqing Wu, Ting Gao, Hai Li, Qing Xie

**Affiliations:** 1 Department of Infectious Diseases, Ruijin Hospital, Shanghai Jiaotong University School of Medicine, Shanghai, China; 2 Discipline of Pathology, Bosch Institute and School of Medical Sciences, University of Sydney, Sydney, New South Wales, Australia; 3 Department of Medicine, Baylor College of Medicine, Houston, Texas, United States of America; 4 Department of Gastroenterology, Renji Hospital, Shanghai Jiaotong University School of Medicine, Shanghai Institution of Digestive Disease, Key Laboratory of Gastroenterology and Hepatology, Ministry of Health, Shanghai, China; CIMA. University of Navarra, Spain

## Abstract

**Background:**

Acute-on-chronic liver failure (ACLF) is an acute deterioration of established liver disease. Blocking the TNF (tumor necrosis factor)/TNFR (tumor necrosis factor receptor) 1 pathway may reduce hepatocyte apoptosis/necrosis, and subsequently decrease mortality during development of ACLF. We demonstrated that a long-acting TNF antagonist (soluble TNF receptor: IgG Fc [sTNFR:IgG-Fc]) prevented/reduced development of acute liver failure by blocking the TNF/TNFR1 (TNFRp55) pathway. However, it is still unclear if sTNFR:IgG-Fc can inhibit hepatocyte damage during development of ACLF.

**Methodology:**

Chronic liver disease (liver fibrosis/cirrhosis) was induced in Wistar rats by repeatedly challenging with human serum albumin (HSA), and confirmed by histopathology. ACLF was induced with D-galactosamine (D-GalN)/lipopolysaccharide (LPS) i.p. in the rats with chronic liver disease. Serum and liver were collected for biochemical, pathological and molecular biological examinations.

**Principal Findings:**

Reduced mortality was observed in sTNFR:IgG-Fc treated ACLF rats, consistent with reduced interleukin (IL)-6 levels in serum and liver, as well as reduced hepatic caspase-3 activity, compared to that of mock treated group. Reduced hepatic damage was confirmed with histopathology in the sTNFR:IgG-Fc treated group, which is consistent with reduced Bcl-2 and Bax, at mRNA and protein levels, but increased hepatocyte proliferation (PCNA). This is also supported by the findings that caspase-3 production was up-regulated significantly in ACLF group compared to the mock treated group. Moreover, up-regulated caspase-3 was inhibited following sTNFR:IgG-Fc treatment. Finally, there was up-regulation of hepatic IL-22R in sTNFR:IgG-Fc treated ACLF rats.

**Conclusions:**

sTNFR:IgG-Fc improved survival rate during development of ACLF *via* ameliorating liver injury with a potential therapeutic value.

## Introduction

Acute-on-chronic liver failure (ACLF) is defined as liver decompensation that develops rapidly secondary to chronic liver disease [1], [2]. The pathological feature of ACLF presents with acute hepatocyte necrosis and apoptosis in the presence of chronic liver failure [1], [2]. ACLF is very common in chronic hepatitis B patients with high mortality and morbidity in China. Understanding the underlying mechanism of hepatic damage during development of ACLF is important in generating novel drugs to prevent/treat such a devastating disease.

It has been demonstrated in our previous study that the TNF-mediated excessive immune cascade response resulted in massive hepatocyte apoptosis and impaired hepatocyte proliferation during the development of acute liver failure [3]. TNFRp55 (a membrane-bound receptor) is important in acute hepatocyte necrosis/apoptosis [4], because TNF contributes to damage of hepatocyte (necrosis/apoptosis) in ACLF *via* the TNF/TNFRp55 signaling pathway [4], [5]. Our previous study had demonstrated that a soluble TNF receptor: IgG-Fc fusion protein (sTNFR:IgG-Fc) reduced acute liver damage/failure in mice by blocking membrane-bound TNFRp55 and the TNFRp55 signaling pathway [6], [7]. However, it is unclear if sTNFR:IgG-Fc is also able to reduce/prevent the development of ACLF. In the current study, we developed an ACLF model that mimics human ACLF in clinical and laboratory presentations. According to the “second hit” theory, liver fibrosis was induced with human serum albumin and followed by injection of D-galactosamine/lipopolysaccharide (LPS) [3], [4], [8], resulting in ACLF. The efficacy of sTNFR:IgG-Fc in ACLF and the TNF/TNFR p55 signaling pathway are investigated in the ACLF model. The data obtained from this study provide some useful information in the treatment of ACLF.

## Materials and Methods

### Ethics Statement

All animal care and experimental procedures complied with the *guidelines for the Care and Use of Laboratory Animals,* formulated by the Ministry of Science and Technology of the People’s Republic of China, and were approved by the *Ethical Committee on Animal Experiments at Shanghai Jiao Tong University*.

### Animals

SPF Wistar rats, weighing 150–160 g, were obtained from *the Shanghai SLAC Laboratory Animal Co. Ltd* (Shanghai, China). Animals were accommodated for one week before the experiment at 23°C in a 12∶12 hours light-dark cycle with food and water *ad libitum*.

### Induction of ACLF in Rats

Rats were injected s.c. with human serum albumin (HSA) 25 mg/kg (Baxter, Illinois, USA) emulsified in Freund’s complete adjuvant at days 0, 14, 24 and 34. The sensitized status was confirmed by detecting serum anti-HSA antibody level from the challenged rats 10 days after the fourth immunization. The sensitized animals further received HSA i.v. twice weekly for a further six weeks. In the first week, the rats received HSA i.v. 2.5 mg/rat, followed by 3.0 mg/rat for the second dose. In the second week, the rats received twice of HSA i.v. challenged 3.5 and 4.0 mg/rat. For the next four weeks, the rats received HSA i.v. 4.5 mg/rat each week. Finally, ACLF was induced by intraperitoneal injection of D-galactosamine (D-GalN) (400 mg/kg) and lipopolysaccharide (LPS) (100 µg/kg) (Sigma-Aldrich, St. Louis, MO USA). There were 8 rats for each ACLF challenged or ACLF plus sTNFR:IgG-Fc treated group, but only 6 rats for the control. The animals were sacrificed by lethal dose of barbital.

### The Function of sTNFR:IgG-Fc

To determine whether sTNFR:IgG-Fc can prevent the development of ACLF, chronic liver cirrhotic rats were treated with sTNFR:IgG-Fc or vehicle intraperitoneal (i.p.) 18 hours prior to the induction of acute liver failure by D-GalN/LPS. The general condition and survival time of the animals were monitored for 12 hours. The challenged rats were sacrificed at different time points (4, 8 and 12 hour) and serum was collected for biochemical analysis and ELISA. Part of the liver was stored in liquid nitrogen for qRT-PCR; whereas another part was fixed in 10% formalin for histopathology.

Furthermore, to determine if sTNFR:IgG-Fc can be used to treat individuals with ACLF, sTNFR:IgG-Fc was given at 0, 1, 2, 4, 8 and 12 hours after D-GalN/LPS injection (i.e. in the early stages of ACLF development).

### The Liver Function Assay

Serum transaminase (ALT) is considered to be the gold standard for detection of liver injury; whereas serum aspartate transaminase (AST) is less specific. Serum ALT and AST were determined by a standard autoanalyzer (Beckman Coulter, Fullerton, California, USA). Prothrombin time (PT), time for the plasma clotting, is also used to determine liver function, as liver functional abnormality results in deficiency of coagulation factors.

### Hepatic IL-6 and IL-22 Production

IL-6 is a classic proinflammatory cytokine, whereas IL-22 plays an important role in the protection of the liver and promotes liver tissue regeneration following liver injury [9]–[11]. We have shown previously that IL-22 may provide protection in liver inflammation/fibrosis during CHB infection [12]. Furthermore, IL-22R is expressed strongly in the liver and other tissues in response to IL-22 stimulation [13], [14]. Thus, serum IL-6 and IL-22 levels were determined by ELISA, according to the manufacturer’s instructions (B&D Biosciences, San Diego, California, USA).

### Hepatic Caspase-3

Caspases are crucial mediators of apoptosis; caspase-3 is a frequently activated death protease [15]. For detection of apoptosis, caspase-3 activity was quantified using caspase colorimetric assay kits (Beyotime Institute of Biotechnology, Shanghai, China). In brief, tissue was homogenized in ice-cold lysis buffer for 30 seconds using a homogenizer. Supernatants were collected by centrifugation for 10 minutes at 10,000×g at 4°C. Caspase-3 activity was determined spectrophotometrically, monitoring the chromophore p-nitroanilide (pNA) after cleavage from the labeled substrates of caspases Ac-DEVD-pNA. The results were expressed as nanomoles of pNA per minute per milligram of protein. A standard curve was performed for each plate and used to calculate the absolute concentrations of the caspase-3.

### RNA Extraction and Real-time Quantitative PCR

Real-time PCR was performed as previously described [3]. Briefly, total RNA was extracted from specimens using an RNeasy Mini Kit (Qiagen, Maryland, USA). mRNAs were converted to cDNAs using a sensiscript RT Kit (Qiagen, Maryland, USA) and two-step real-time PCR was carried out by using SYBR Green Master Mix (Applied Biosystems, California, USA), according to the manufacturer’s instructions. Primers were developed using Primer Express 2.0 software (Applied Biosystems) and are presented in Table 1.

**Table 1:** Primers sequences.

Gene	Sequence
β-actin	5′-TCTGTGTGGATTGGTGGCTCTA-3′ (forward)
	5′-CTGCTTGCTGATCCACATCTG-3′(reverse)
Bcl-2	5′-GGGATGCCTTTGTGGAACTATA TG-3′ (forward)
	5′-TGAGCAGCGTCTTCAGAGACA-3′ (reverse)
Bax	5′-GACACCTG AGCTGACCTTGGA-3′ (forward)
	5′-GACACTCGCTCAGCTTCTTGGT-3′ (reverse)
PCNA	5′-ACGTCTCCTTAGTGCAGCTTACTCT-3′(forward)
	5′-CGCCCATGGCCAGGTT-3′ (reverse)
IL-22R	5′- CCGGGAGCATATCCAAAAGA-3′ (forward)
	5′- GGGTAGACAATG CTCTAAGAAAATCA-3′ (reverse)

Thermocycler conditions included an initial holding period at 50°C for 2 minutes, then 95°C for 10 minutes, followed by a 2-step PCR program: 95°C for 15 seconds and 60°C for 60 seconds for 40 cycles. Data was quantitatively analyzed on an ABI PRISM 7900 sequence detection system (Applied Biosystems). The GAPDH gene was used as an endogenous control. The amount of gene expression was then calculated as the difference in cycle threshold (ΔCT) between the CT value of the target gene and that of GAPDH.

### Western-blot

Total protein extracted from liver tissue was quantified using the bicinchoninic acid method (Pierce, Rockford, IL, USA). Protein (40 µg) was loaded for electrophoresis through a 10% SDS-polyacrylamide gel and transferred to a PVDF membrane (Millipore, Billerica, MA, USA). The membranes were blocked with 5% dry milk for 1 h in Tris-saline buffer and 0.1% Tween-20 (TBS/Tween-20). After washing in TBS/Tween-20, the membranes were incubated with rabbit anti-mouse BCL2 (1∶2000, Sigma Aldrich) or rabbit anti-mouse β-actin (1∶5000, Sigma Aldrich) overnight at 4°C. After washing with TBS/Tween-20, the membranes were incubated for 1 h at room temperature with HRP-conjugated goat anti-rabbit IgG (1∶2000, Sigma Aldrich, St Louis, MO, USA ), washed with TBS/Tween-20 and the proteins of interest on the membrane were detected with the SuperSignal West Pico chemiluminescent substrate (Pierce, Rockford, IL, USA).

### Histopathology and Immunohistochemistry

Liver tissues were fixed in 10% formalin-fixed and embedded in paraffin after sacrificing, as described previously [3], [16], [17]. For routine histological analysis, 5 µm sections were cut and stained with hematoxylin & eosin (H&E). Histopathology was scored under a light microscope based on the criteria in Table 2 in a double-blind fashion [18]. Using rabbit anti-human/mouse caspase-3 antibody (Cell Signalling Technology, USA), 5 µm sections of paraffin-embedded liver were stained for the detection of apoptosis. The sections were treated with antigen retrieval buffer (pH 9.0, Dako, Australia) at 100°C for 10 min. The sections were incubated with the primary antibody (1∶200) for 1 h at room temperature, washed three times in Tris-buffered saline (TBS), and incubated with Envision-labelled polymer-HRP rabbit antibody (Dako) for 1 h at room temperature. The peroxidase activity was visualised using diaminobenzidine (DAB).

**Table 2:** Histopathological score for liver.

Pathology	0	1	2	3	4
Eosinophilic staining+hepatic sinusoidal congestion	none	<25%	25%–50%	50%–75%	75%–100%
Cellular swelling	none	<25%	>25%	–	–
Cytolysis	none	<25%	>25%	–	–

### Statistical Analysis

Statistical analyses were performed by computer-assisted one-way ANOVA to compare group means. The analyses were performed on the raw data values (parametric analysis) using the least significant differences means comparison procedure. The data were expressed as mean ± SEM. The comparisons with the control groups were assessed using one-tailed tests. Differences where P<0.05 were considered statistically significant.

## Results

### sTNFR:IgG-Fc Improved Survival Rate and Reduced Liver Injury in ACLF

**Figure 1:**
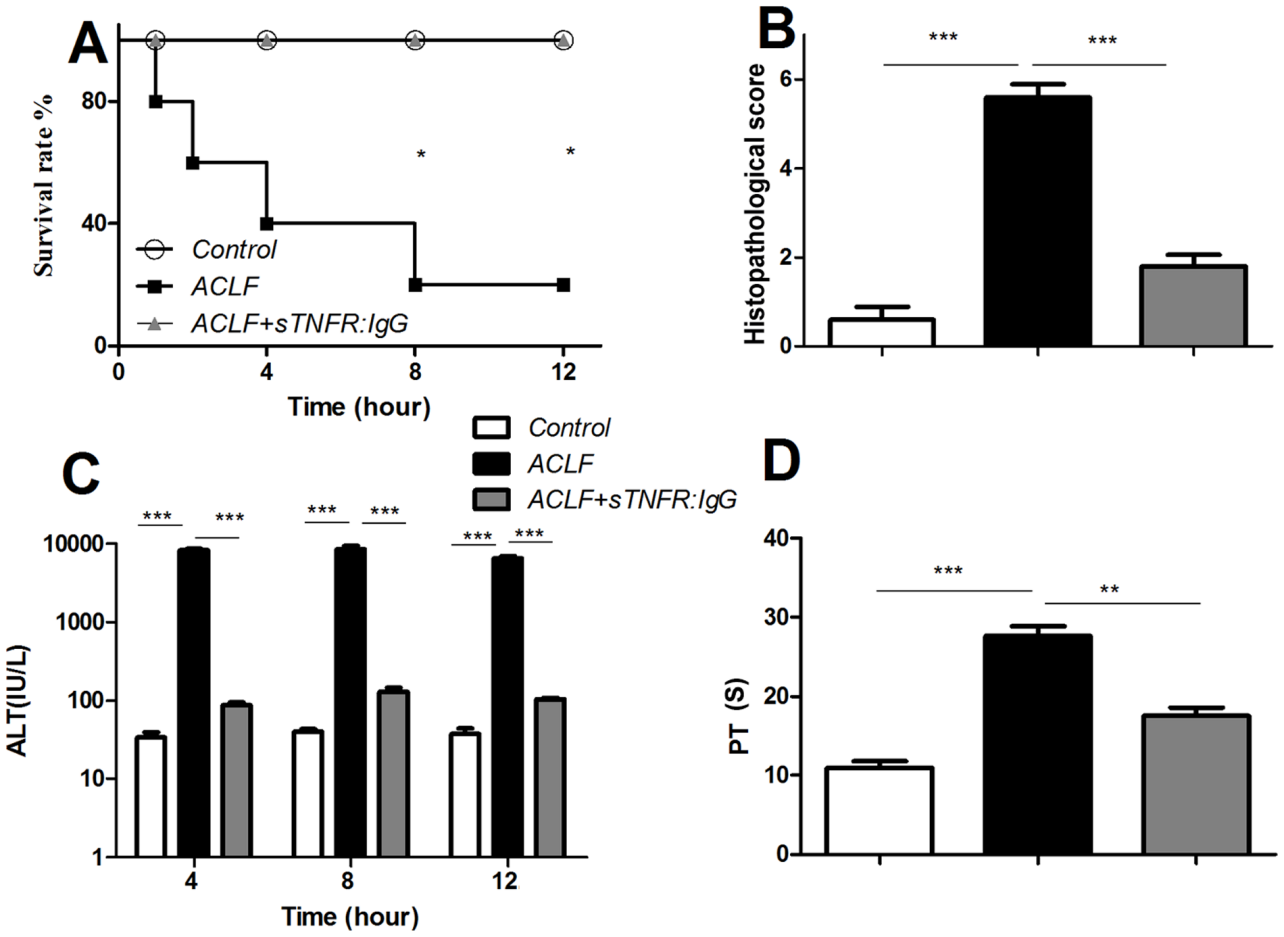
Survival rate of the animals treated with ACLF induction (n = 8) (black square), ACLF with sTNFR:IgG-Fc treatment (n = 8) (grey triangle) and control (n = 6) (white circle) with significant difference (p<0.05) (Figure 1A). Histopathological scores (Figure 1B). No obvious lesion was detected in the control (n = 6) (white bar), high level of lesion was observed in the ACLF (n = 8) (black bar), and significant reduced lesion was observed in the ACLF+sTNFR:IgG-Fc treatment (n = 8) (grey bar). The corresponding pictures are shown in Figure 2 A–C. ALT level was detected in all three groups (Figure 1C). X-axis represents different treatments, Y-axis represents ALT in international units in log scale (IU). As expected, there was constitutive level of ALT in the control (n = 6) (white bar), and ALT was substantially induced in the ACLF liver (n = 8) (black bar), whereas the induced ALT can be inhibited significantly by sTNFR:IgG-Fc treatment (n = 6) (grey bar). Prothrombin time (PT) was determined in the three groups (Figure 1D). X-axis represents different treatments, Y-axis represents PT level. As expected, there was constitutive level of PT in the control (n = 6) (white bar), and PT was substantially induced in the ACLF liver (n = 8) (black bar), whereas the induced PT can be inhibited significantly by sTNFR:IgG-Fc treatment (n = 6) (grey bar). (** P<0.01, ***P<0.001).

**Figure 2:**
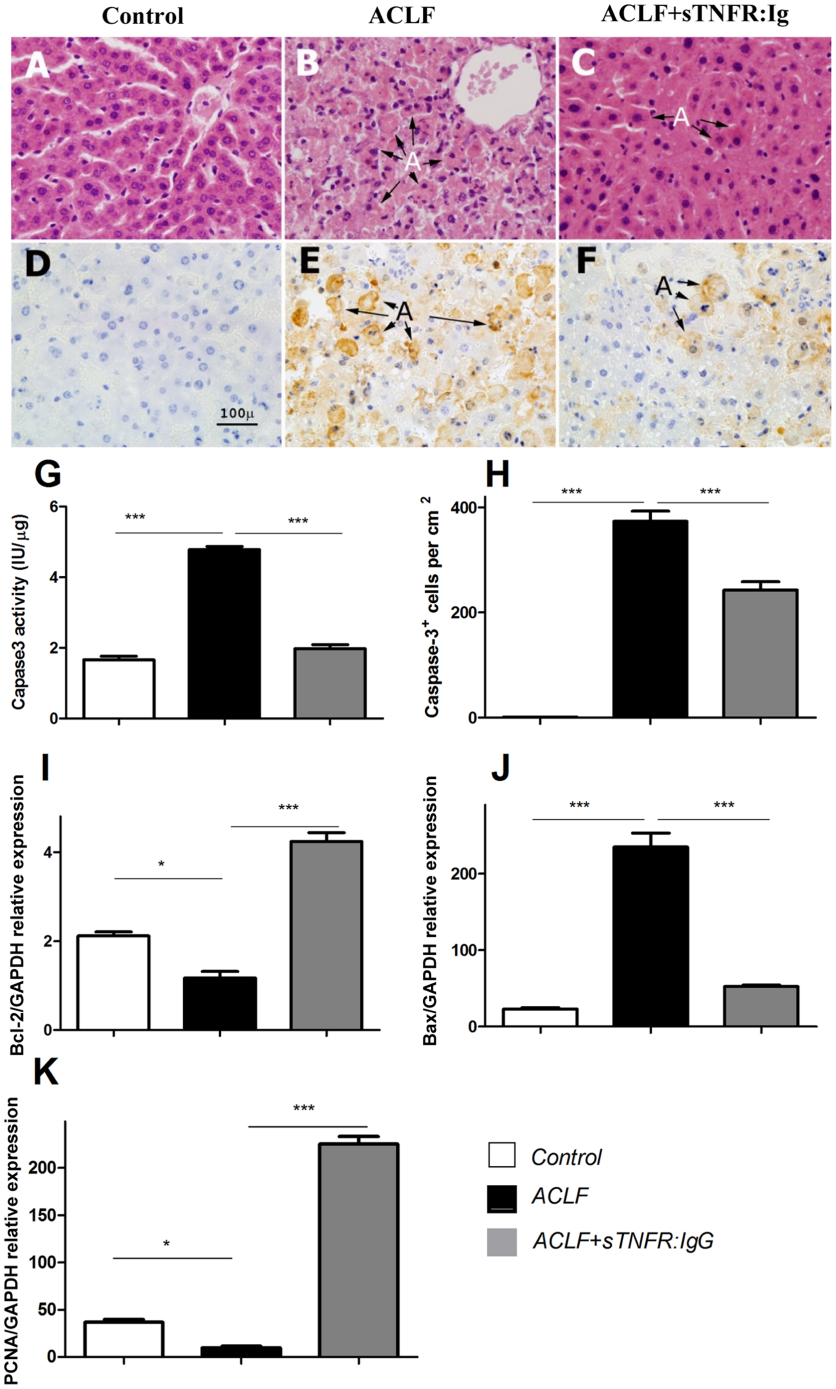
Histopathology of the liver from the control (n = 6) (Figure 2A), ACLF liver (n = 8) (Figure 2B) and ACLF+sTNFR:IgG-Fc treatment (n = 8) (Figure 2 C). Immunohistochemistry of caspase-3 production in the liver with different treatments (Figure 2D, control), ACLF (Figure 2E) and ACLF+sTNFR:IgG-Fc treatment (Figure 2F). Caspase-3 activity detected by the kit (Figure 2G) in the liver from the different treatments. Quantification of immunohistochemistry of caspase-3^+^ cells (Figure 2H) in the liver with different treatments. Y axis represents the relative mRNA expression of Bcl-2, Bax or PCNA compared to that of housekeeping gene GAPDH (Figure 2 I–K). The data are the average with SEM of all the samples in each group. X-axis represents different treatments [the control (n = 6) (white bar), the ACLF liver (n = 8) (black bar), and sTNFR:IgG-Fc treatment (n = 6) (grey bar)]. Y-axis represents levels of caspase-3 activity, caspase-3^+^ cells, or respective gene expression. (** P<0.01, ***P<0.001).

The survival rates in ACLF rats pre-treated with sTNFR:IgG-Fc or vehicle was 100% or 20% with significant difference (P<0.05), i.e. sTNFR:IgG-Fc treatment prevented death (Figure 1A). Histopathology showed that substantial liver damage in ACLF rats was significantly attenuated with sTNFR:IgG-Fc treatment (Figure 1B), compared to that of vehicle treated or normal control (1.8±0.29 vs 5.6±0.25 *vs* 0.6±0.29, p<0.001). The typical histopathology is presented in the figure 2 (A–C). This is also consistent with the findings that ALT (6533±359.59 vs 37.33±5.53 or 105±3.19, p<0.001) and PT (27.6±1.30 vs 10.93±0.86 or 17.53±1.07, p<0.001) decreased by 98% or 36.5% in sTNFR:IgG-Fc treated ACLF rats, respectively, compared to that of vehicle treatment only (Figure 1C, 1D).

The chronic liver damage was confirmed by histopathology revealed substantial necrosis of hepatocytes with regenerative nodules and intact fibrous septum of the liver in the rat model of ACLF(Figure S1A), compared to that of control (Figure S1B), consistent increased ALT levels but not PT (Figure S1C and 1D).

### Histopathology and Immunohistochemistry

No abnormality was observed in the liver from the control (Figure 2A). Substantial damage was detected in the ACLF liver (Figure 2B), with noticeable destruction of architecture; a large number of apoptotic (indicated by A) and necrotic cells was observed in the ACLF liver. In addition, infiltrating cells and congested red blood cells in the sinusoids were presented. On the other hand, in the sTNFR:IgG-Fc treated rats, the hepatocytes seemed to be relatively healthy, with rich eosinophilic cytoplasm and fewer apoptotic cells, as compared to those from ACLF rats (Figure 2C). Notwithstanding, it was observed that hepatocytes were swollen and undergoing apoptosis, sinusoids were congested, and leucocytes were infiltrating into the liver from sTNFR:IgG-Fc treated rats, compared to that of the control.

To confirm apoptotic changes in the liver, caspase-3 activity was determined (Figure 2G). There was constitutive level of caspase-3 in the control liver, which can be up-regulated significantly (1.67±0.10 vs 4.78±0.83, p<0.001) in the ACLF liver. However, such induction can be inhibited by sTNFR:IgG-Fc (4.78±0.83 vs 1.98±0.11, p<0.001). Furthermore, caspase-3 was also confirmed by immunohistochemistry (Figure 2 D–F, and 2H). There were few caspase-3^+^ cells in the control (Figure 2D and H). However, substantial caspase-3^+^ cells were presented in the ACLF liver (Figure 2E and H), which was ∼400-fold higher than that of the control (0.5±0.21 vs 373±20.19, p<0.001). sTNFR:IgG-Fc treatment reduced caspase-3^+^ cells significantly (Figure 2F and H) (373±20.19 vs 243±16.30, p<0.001), which was ∼30% reduction.

Bcl-2 and Bax are associated with apoptosis [17], whereas PCNA reflects cellular proliferation during liver damage. Thus these three molecules were investigated in our ACLF model with/without sTNFR:IgG-Fc treatment, using real-time PCR (Figure 2 I–K).

The results showed that the expression of Bax mRNA (Figure 2J) was increased by >10-fold (22.72±3.17 vs 234±18.64, p<0.001), but Bcl-2 mRNA (2.12±0.09 vs 1.17±0.15, p<0.05) (Figure 2I) and PCNA mRNA (36.87±2.65 vs 9.78±1.47 p<0.05) (Figure 2K) were decreased significantly in the ACLF liver. The induction of Bax mRNA (234±18.64 vs 52.32±1.91, p<0.001) and reduction of Bcl-2 (1.17±0.15 vs 4.24±0.20, p<0.001) and PCNA (9.78±1.47 vs 225.32±7.94, p<0.001) from the ALCF liver, however, can be reversed by sTNFR:IgG-Fc treatment.

### sTNFR:IgG-Fc Modified IL-6, IL-22 and IL-22R Production in ACLF Rats

**Figure 3:**
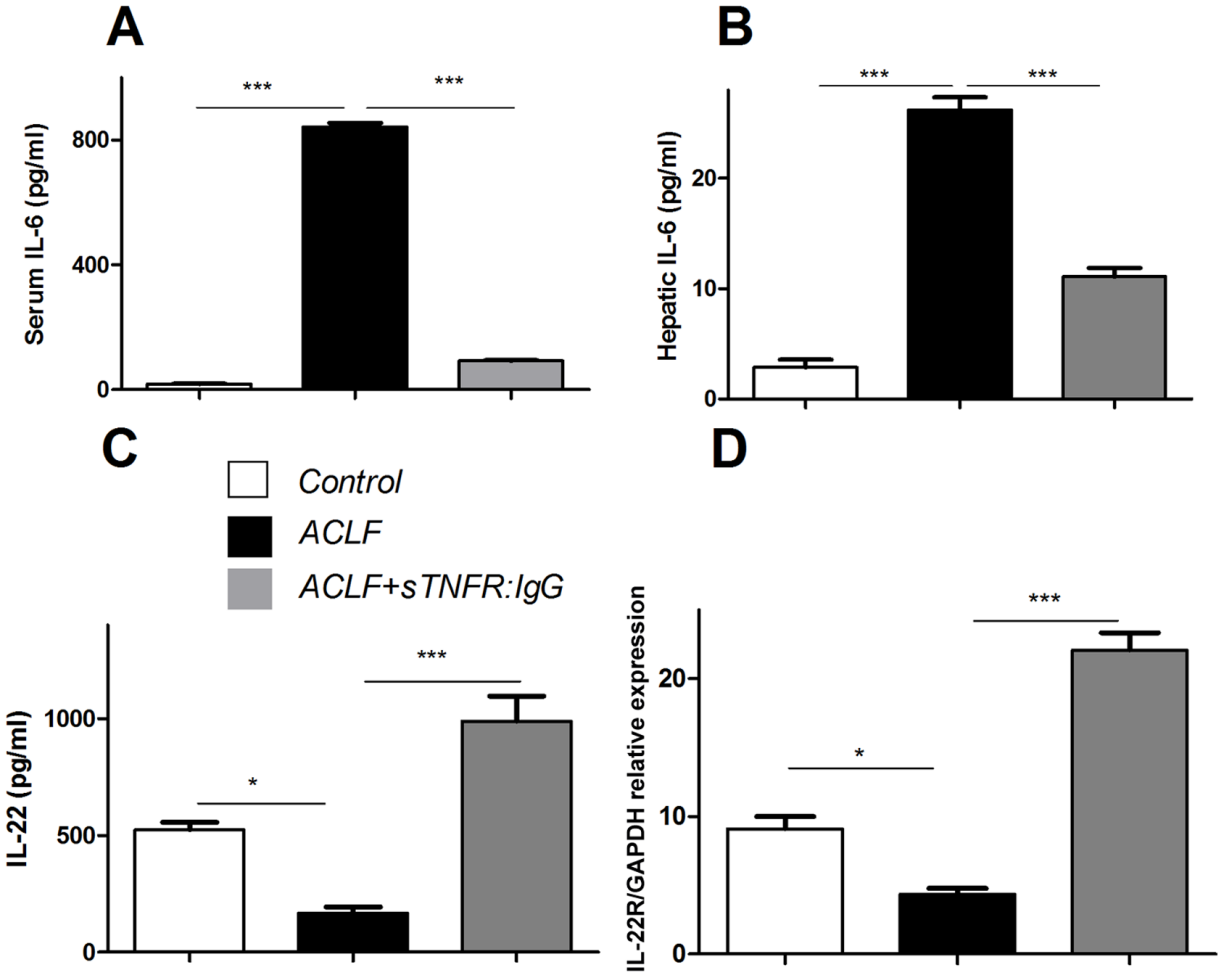
The levels of IL-6 in the serum (Figure 3A) and the liver (Figure 3B), IL-22 in the liver (Figure 3C) and IL-22R in the liver (Figure 3D) were detected. X-axis represents different treatments [the control (n = 6) (white bar), the ACLF liver (n = 8) (black bar), and sTNFR:IgG-Fc treatment (n = 6) (grey bar)]. Y-axis represents the levels of respective cytokine. (** P<0.01, ***P<0.001).

To evaluate the inflammatory status of liver, IL-6 was used as a marker. It was found that IL-6 was increased substantially in the serum (27.86±0.66 vs 842±12.94, p<0.001) or liver (2.87±0.70 vs 26.20±1.15, p<0.001) from ACLF animals (Figure 3A and B). Such induction, however, can be inhibited with sTNFR:IgG-Fc treatment, which reduced by ∼90% and ∼60% in the serum (842±12.94 vs 91.90±1.58, p<0.001) or liver (26.20±1.15 vs 11.10±0.81, p<0.001), respectively, compared to that of vehicle treated ACLF group (Figure 3A and B).

It has been demonstrated that IL-22 provides a hepato-protection in our previous report [12]. Thus, IL-22 production in liver was determined. There was constitutive IL-22 expression, but was suppressed significantly (523±33.65 vs 167±27.80, p<0.05) in the ACLF liver (Figure 3C). However, sTNFR:IgG-Fc treatment up-regulated significantly (167±27.80 vs 988±109.61, p<0.01) IL-22 production in the liver (Figure 3C). Similar pattern of IL-22R expression was also detected in the ACLF with/without sTNFR:IgG-Fc treatment (9.09±0.92 vs 4.37±0.42 vs 22.08±1.26, p<0.001). (Figure 3D).

### Bax, Bcl-2 and PCNA Production, Determined by Western Blot

**Figure 4:**
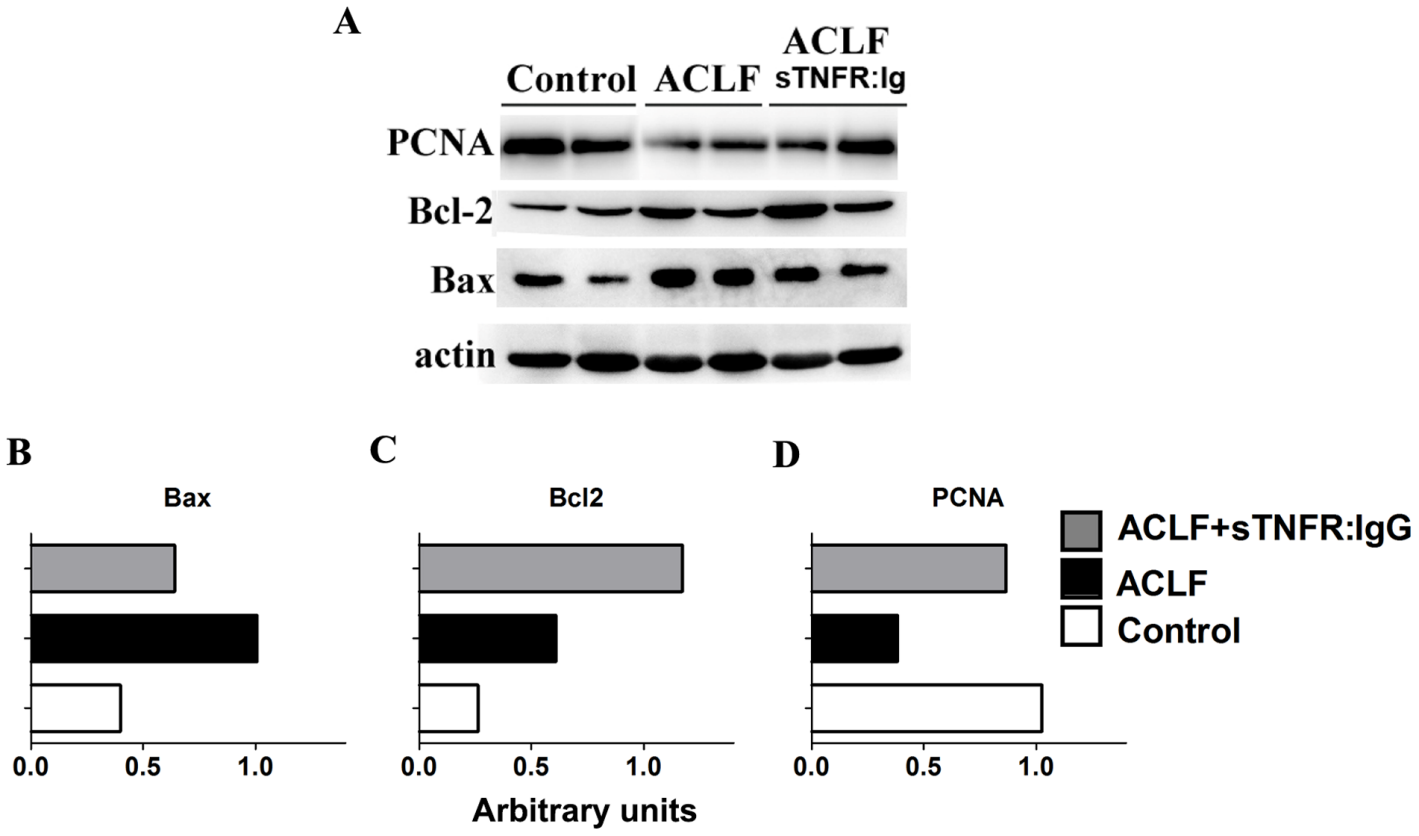
Protein levels of Bax, Bcl-2 and PCNA were detected by Western blot (Figure 4A) and quantified individually Bax (Figure 4B), Bcl-2 (Figure 4C) and PCNA (Figure 4D). Figure 4B–D: X axis represents the relative gray values of Western blot bands of Bcl-2, Bax or PCNA compared to that of house keeping protein beta-actin.

It has been demonstrated that Bax has pro-apoptotic and Bcl-2 has anti-apoptotic properties during chronic liver injury [24]. The protein production of Bax, Bcl-2 and PCNA was confirmed, using Western blot (Figure 4A). Consistent with mRNA expression, similar patterns of Western blot was detected at the protein level, i.e. the expression of Bax protein (Figure 4B) was also reduced in sTNFR:IgG-Fc group compared with ACLF group, whereas the level of Bcl-2 (Figure 4C) and PCNA (Figure 4D) protein was increased. The reduction of Bax and induction of Bcl-2 and PCNA, however, were reversed by sTNFR:IgG-Fc treatment (Figure 4).

## Discussion

In this study, we have developed a rat model of ACLF by immune system-induced liver cirrhosis induced with HSA, which was then exacerbated with D-galactosamine/LPS to establish ACLF. Prior to acute attack, the chronic liver damage was confirmed by histopathology and revealed substantial necrosis of hepatocytes with regenerative nodules and intact fibrous septa of the liver in the rat model of ACLF, consistent with biochemistry.

Based on the criteria from *The Asian Pacific Association for the Study of the Liver* (APASL), the definition of acute on chronic liver failure (ACLF) in human is that chronic liver disease with acute hepatic attack, resulting in rapid liver function deterioration accompanied by high mortality in short term [2]. In our current animal model, it has been demonstrated that liver fibrosis/early stage cirrhosis is induced with multiple human albumin challenge in 6 weeks, resulting in chronic liver damage. Subsequent Gal/LPS acute attack/hit contributes to focal necrosis, piece meal necrosis, red blood cell influx within 4 hours, and sub-massive hepatic necrosis within 12 hours, which is supported by the findings that the level of ALT/AST is increased substantially, starts at 4 hours and peaks at 8 hours; bilirubin, on the other hand, is increased continuously till death of the animals (data not shown). Finally, high rate of mortality is detected within short period of Gal/LPS attack, starting at 10 hours and reaching 80% at 24 hours. Thus, our current model reflects well with the criteria of ACLF in human. Such a model allows us to explore the pathophysiology of ACLF and to determine the efficacy of novel drugs.

It has been demonstrated that TNF plays an important role in the pathogenesis of liver failure [3]–[6]. In the current study we demonstrate that sTNFR:IgG-Fc improved survival rate substantially in ACLF rats, which is consistent with reduced clinical signs, inflammation and necrosis in livers. This is consistent with our previous findings in acute liver failure [6]. The main advantages of sTNFR:IgG-Fc are that it has a long half-life (up to one week) [7] and is effective in comparison to TNF antibody treatment in ACLF. Our previous studies showed that anti-TNF antibodies or soluble TNFRp55 were unable to block the TNF/TNFRp55 pathway, and reduce the mortality of animals with acute liver injury. Despite the failure of antibodies directed to TNF/TNFRp55, effective blocking of this pathway remains a target for the reduction of hepatocyte damage and subsequent liver failure. Logically, membrane-bound TNFRp55 is an important target for developing drugs against acute necrosis of hepatocytes. Moreover, sTNFR:IgG-Fc also improved survival rate of ACLF rats, which is supported by the findings that sTNFR:IgG-Fc reduced morbidity significantly after induction of ACLF.

In ACLF, the systemic inflammatory response is closely tied to its clinical outcomes [19]. The final common pathway leading to acute deterioration of liver function and multi-organ failure appears to be an exaggerated activation of systemic inflammation [20]. In the current study, we found that the level of IL-6, an important inflammatory cytokine in liver inflammation, was significantly reduced in rats treated with sTNFR:IgG-Fc. IL-22 is protective for hepatocytes and also functions synergistically with TNF, IL-1β and IL-17 [11], [12], [21]. Expression of IL-22R was increased in the liver, while the level of IL-22 in the serum of rats following sTNFR:IgG-Fc treatment was correspondingly raised in the current study. This suggests that sTNFR:IgG-Fc protects against liver injury *via* the IL-22 signaling pathway. This observation leads us to hypothesize that sTNFR:IgG-Fc and IL-22 combined therapy may achieve more effective improvement in ACLF through the sTNFR:IgG-Fc-IL-22R-IL-22 pathway, which is currently being investigated.

Furthermore, our current chronic liver condition is induced by human albumin *via* immunological pathways, which maybe closely relate to patients with chronic hepatitis B viral infection, from a pathophysiological point of view. On the other hand, acetaminophen-induced drug toxicity [22] and carbon tetrachloride chemical damage of liver [23] are, rather, hepatotoxic mediated chronic liver condition. We understand that the final stage of any chronic liver damage will lead to terminal liver decompensation. Moreover, it is well known that the common triggers for ACLF are infection and alcohol toxicity in patients with chronic liver disease, resulting in liver decompensation. Our current model seems to more reliably mimic decompensated human chronic viral hepatitis, and may be useful for development of pharmaceutical agents.

In summary, we have established a useful rat model of human ACLF. Using this model, it has been demonstrated that sTNFR:IgG-Fc significantly improved survival rate, liver function and decreased inflammation and hepatocytes apoptosis, possibly via TNF/TNFRp55. This information might provide a therapeutic target for the treatment of ACLF.

## Supporting Information

Figure S1 **Histopathology of control liver (Figure S1A), and chronic liver disease (Figure S1B).** ALT (Figure S1C) and PT (Figure S1D) levels of the control *vs* chronic liver. Thin fibrous septa with dissecting nodules were observed. Large number of infiltrating leucocytes was presented in the portal region. No regenerative nodule was observed in the liver, suggesting it was early cirrhosis. Furthermore, there was more than 2-fold increased ALT in the challenged group, compared to that of the mock challenged group, supporting liver damage. As expected, there was no significant difference of PT between cirrhotic and mock challenged groups, suggesting that it was not liver failure.
(TIF)
