# Supplementary figures and images for: Amelioration of Liver Injury by Continuously Targeted Intervention against TNFRp55 in Rats with Acute-on-Chronic Liver Failure
Source: PLoS One. 2013 Jul 16;8(7):e68757. doi: 10.1371/journal.pone.0068757 (PMC3712937; doi:10.1371/journal.pone.0068757)

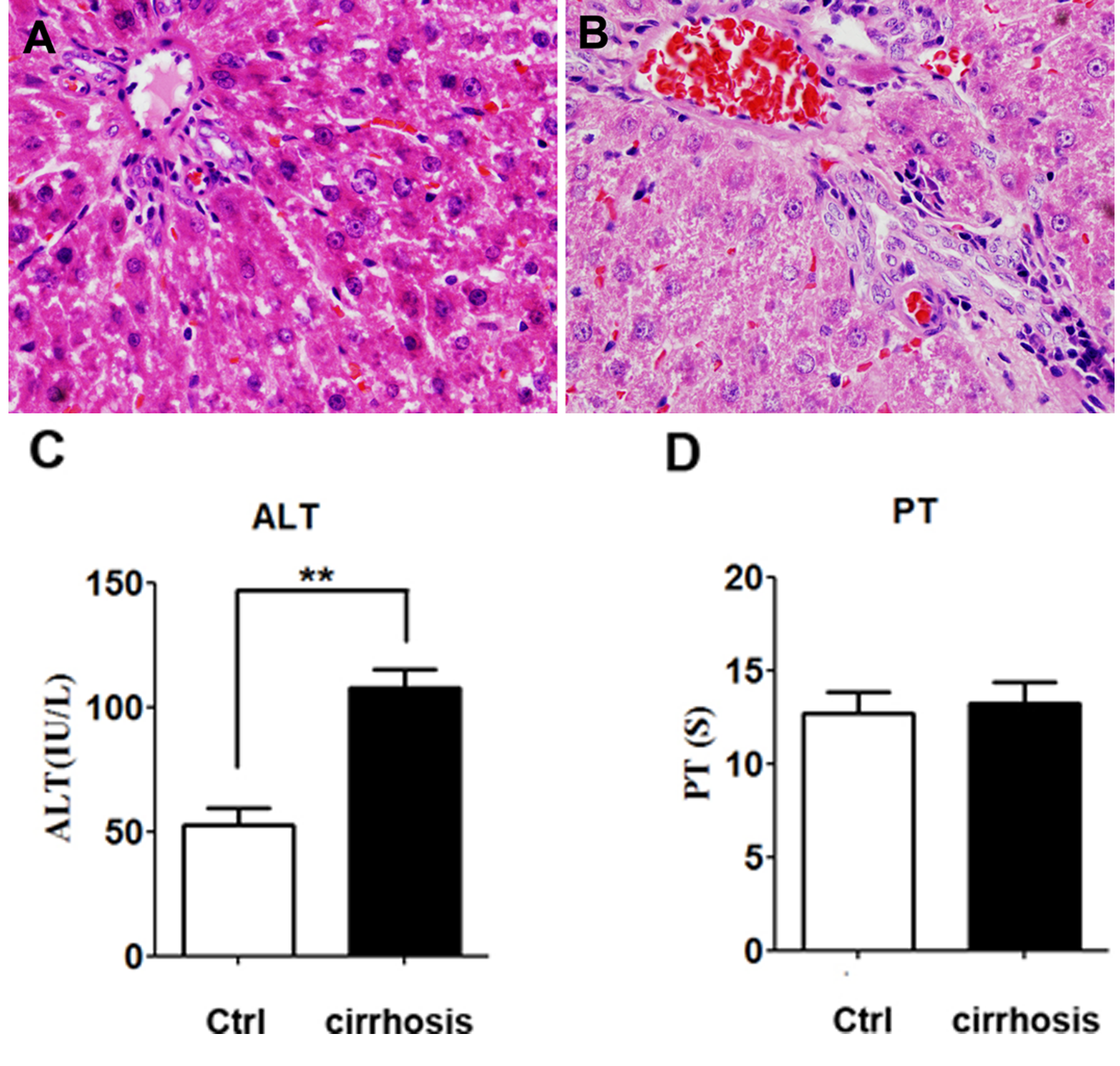

Supplement: Figure S1 — Histopathology of control liver (Figure S1A), and chronic liver disease (Figure S1B). ALT (Figure S1C) and PT (Figure S1D) levels of the control vs chronic liver. Thin fibrous septa with dissecting nodules were observed. Large number of infiltrating leucocytes was presented in the portal region. No regenerative nodule was observed in the liver, suggesting it was early cirrhosis. Furthermore, there was more than 2-fold increased ALT in the challenged group, compared to that of the mock challenged group, supporting liver damage. As expected, there was no significant difference of PT between cirrhotic and mock challenged groups, suggesting that it was not liver failure. (TIF) [file pone.0068757.s001.tif]
